# Scar heterogeneity in hypertrophic cardiomyopathy. Comparison with chronic myocardial infarction and normal subjects

**DOI:** 10.1186/1532-429X-16-S1-P369

**Published:** 2014-01-16

**Authors:** Pier Filippo Vianello, Luca Olivotti, Eva Sammut, Reza Razavi, Eike Nagel, Amedeo Chiribiri

**Affiliations:** 1 Cardiovascular Imaging, King's College London, London, UK; 2 Internal Medicine, University of Genoa, Genoa, Italy

## Background

Recent evidence has given importance to heterogenous scar areas (HSA) in the identification of patients receiving appropriate implantable-cardioverter-defibrillator (ICD) therapy (Rayatzadeh, JCMR 2013;15:31). Late gadolinium enhancement (LGE) CMR has the potential of becoming one of the main tools for risk stratification in patients affected by hypertrophic cardiomyopathy (HCM). While visual analysis of LGE is commonly used for clinical reporting, quantitative LGE analysis has been used in several clinical studies. The aim of this study was to evaluate the presence of HSA in patients with HCM and to compare the findings in this group with patients with myocardial infarct (MI) and subject with normal CMR scan.

## Methods

72 consecutive patients with known HCM, 10 patients with normal CMR findings and no history of myocardial infarction or HC, and 12 patients with previous MI and preserved global left ventricular function (EF > 55%) were included. Scar size was determined using thresholds of 2 and 5 SD above remote normal myocardium and by visual assessment. HSA was defined as the region between 2 and 5 SD.

## Results

Out of the 94 patients in this study there was significant accordance between visual and quantitative assessment for LGE. The amount of HSA was significantly higher in HCM (27.9% ± 9%) than in MI (18.6% ± 4.7%; p < 0.01) and in normal subjects (15.1% ± 6.0%; p < 0.01). No significant differences in the HSA were observed between MI and normal groups (p = 0.67). Moreover a linear correlation between the amount of HSA and the amount of LGE volume detected at 5 SD threshold was found.

## Conclusions

Our results confirm the potential usefulness of HSA in patients with HCM. Differently from patients with previous MI and normal LV ejection fraction - where LGE constitutes a discrete and localised lesion - and differently from subject with normal CMR scan, patient with HCM has significant amounts of HSA on quantitative analysis. Although more studies will be needed to clarify the potential for a clinical use of HSA, the most likely pathophysiological substrate to explain our findings is a direct relationship between HSA and interstitial fibrosis. If this is confirmed, HSA could represent a novel predictor of adverse outcomes in HCM patients and help for improved risk stratification in a clinical setting.

## Funding

The Centre of Excellence in Medical Engineering is funded by the Wellcome Trust and EPSRC under grant number WT 088641/Z/09/Z. The authors acknowledge financial support from the Department of Health via the National Institute for Health Research (NIHR) comprehensive Biomedical Research Centre award to Guy's and St Thomas' NHS Foundation Trust in partnership with King's College London.

